# Abdominal Compartment Syndrome Secondary to Delayed Retroperitoneal Hemorrhage After Renal Biopsy: A Case Report

**DOI:** 10.7759/cureus.82223

**Published:** 2025-04-14

**Authors:** Koichiro Isa, Hiromu Okano, Misa Kitamura, Satoru Sekiya, Hiroshi Okamoto

**Affiliations:** 1 Department of Critical Care Medicine, St. Luke's International Hospital, Tokyo, JPN; 2 Department of Social Medical Sciences, Graduate School of Medicine, International University of Health and Welfare, Tokyo, JPN; 3 Department of Internal Medicine, St. Luke's International Hospital, Tokyo, JPN

**Keywords:** abdominal compartment syndrome (acs), interventional radiology guided embolization, open surgical decompression, renal biopsy complication, retroperitoneal space hemorrhage

## Abstract

Abdominal compartment syndrome (ACS) is a severe clinical condition characterized by increased intra-abdominal pressure, potentially leading to organ dysfunction and high mortality. This report describes a rare case of ACS resulting from delayed retroperitoneal hemorrhage following a renal biopsy. The patient was successfully managed through repeated interventional radiology (IVR) embolization procedures, followed by prompt surgical evacuation of the hematoma. This case underscores the critical role of coordinated intervention between IVR and surgical teams in achieving hemostasis and controlling intra-abdominal hypertension. The collaborative approach highlights the importance of timely, multidisciplinary decision-making in managing biopsy-related hemorrhagic complications, particularly in high-risk patients with coagulopathies.

## Introduction

Abdominal compartment syndrome (ACS) is a clinical condition characterized by sustained intra-abdominal pressure exceeding 20 mmHg, leading to dysfunction of abdominal and systemic organs [1]. Delayed diagnosis and treatment can result in severe complications and high mortality. Common causes of ACS include trauma, acute pancreatitis, extensive abdominal surgery, massive fluid resuscitation, and severe sepsis [2].

ACS secondary to retroperitoneal hemorrhage after renal biopsy is exceedingly rare, with only a few reported cases [3]. Renal biopsy is typically a safe diagnostic procedure, but it can occasionally lead to significant bleeding. Although most biopsy-related bleeding episodes resolve spontaneously, approximately 0.9% require transfusion or active intervention [4]. Patients with chronic liver diseases, such as cirrhosis, are at higher risk due to coagulation disorders, warranting careful attention [5].

Management of ACS caused by retroperitoneal hemorrhage following renal biopsy requires careful timing of interventional radiology (IVR) embolization and surgical decompression. Delayed treatment can rapidly exacerbate organ dysfunction, with mortality rates reaching up to 75% [6]. However, no standardized hemostatic strategy for biopsy-related hemorrhage has been established, necessitating individualized therapeutic approaches for each patient.

Previous reports have described cases of retroperitoneal hemorrhage following renal biopsy, including one in which surgical hematoma evacuation was performed without prior IVR embolization due to the absence of active bleeding [3]. In contrast, our case required multiple sessions of IVR embolization due to persistent hemorrhage before surgical decompression could be performed.

In this report, we present a rare case of ACS secondary to biopsy-induced retroperitoneal hemorrhage in a coagulopathic patient, successfully managed through repeated IVR procedures and prompt surgical intervention. We aim to propose management strategies for complications following renal biopsy.

## Case presentation

A 53-year-old male patient with minimal prior medical contact presented to the emergency department complaining of progressive general fatigue, dyspnea, abdominal distension, and diarrhea over a two-week period. He reported chronic heavy alcohol consumption of approximately 2 L of beer daily (equivalent to 80 g of pure alcohol).

Upon initial examination, the patient was conscious and alert but exhibited marked jaundice, bilateral lower limb edema, prominent ascites, and pronounced abdominal distension. Laboratory tests showed acute kidney injury (serum creatinine: 7.78 mg/dL), liver dysfunction (total bilirubin: 3.00 mg/dL, direct bilirubin: 2.51 mg/dL), anemia (hemoglobin: 8.30 g/dL), markedly elevated inflammatory markers (C-reactive protein: 16.7 mg/dL), and significant coagulopathy (prothrombin time-international normalized ratio: 2.45, activated partial thromboplastin time: 60.2 s, fibrinogen: 604 mg/dL) (Table 1).

**Table 1:** Blood test at the admission, showing signs of acute kidney injury, liver dysfunction, anemia, elevated inflammatory markers, and significant coagulopathy

Test item	Result	Unit	Reference range
White blood cell count	15.0	10^3^/μL	3.30-8.60
Red blood cell count	4.86	10^6^/μL	4.35-5.55
Hemoglobin	8.30	g/dL	13.7-16.8
Hematocrit	43.6	%	40.7-50.1
Platelet count	16.5	10^4^/μL	15.8-34.8
Albumin	3.50	g/dL	4.10-5.10
Blood urea nitrogen	103	mg/dL	8.0-20.0
Creatinine	7.78	mg/dL	0.65-1.07
Aspartate aminotransferase	38.0	U/L	13.0-30.0
Alanine transaminase	17.0	U/L	10.0-42.0
Sodium	124	mEq/L	138-145
Potassium	3.6	mEq/L	3.6-4.8
Chloride	89	mEq/L	101-108
C-reactive protein	16.7	mg/dL	-0.14
Prothrombin Time-International Normalized Ratio	2.45	-	0.80-1.20
Activated Partial Thromboplastin Time	60.2	seconds	23.0-40.0
Fibrinogen	604	mg/dL	150-400

**Figure 1:**
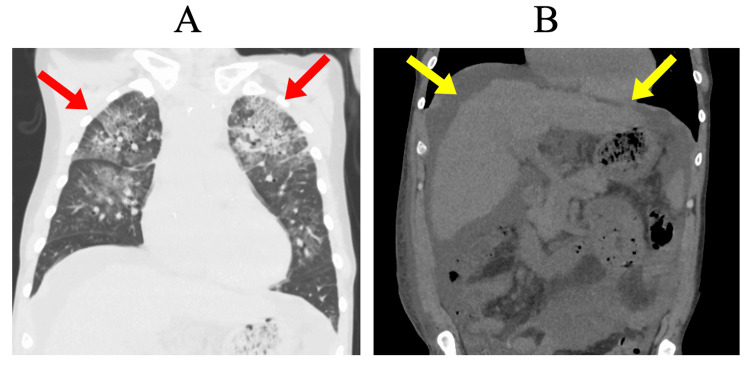
Non-contrast chest and abdominal CT on the initial examination (A) Non-contrast chest CT showing pulmonary infiltrates (red arrows). (B) Non-contrast abdominal CT showing liver cirrhosis characterized by irregular and nodular liver margins (yellow arrows) and ascites. CT, computed tomography.

Computed tomography (CT) confirmed liver cirrhosis, considerable ascites, and pulmonary infiltrates consistent with congestive heart failure and acute kidney injury (Figure 1).

**Figure 2:**
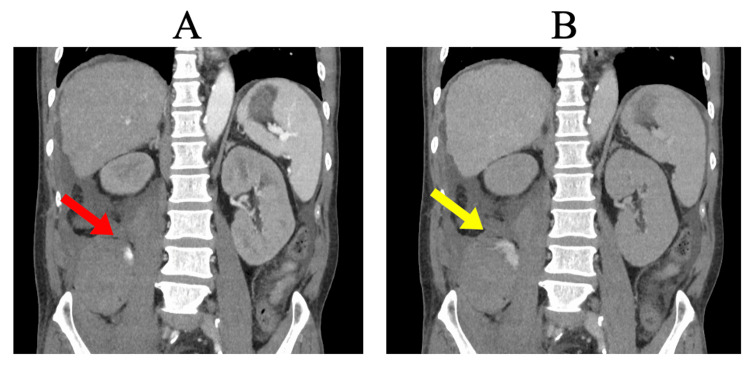
Contrast-enhanced abdominal CT six days after renal biopsy (A) Arterial phase (40 s post-contrast) showing active retroperitoneal bleeding from the right lumbar artery (red arrow). (B) Delayed phase (100 s post-contrast) showing persistent contrast extravasation in the retroperitoneum (yellow arrow). CT, computed tomography.

Initially, conservative management was implemented, including fluid restriction, diuretics, and hemodialysis, aimed at improving renal and cardiac function. However, renal impairment persisted despite these measures. Consequently, an ultrasound-guided renal biopsy was performed on day 27 to evaluate suspected underlying glomerulonephritis. A total of four core specimens were obtained using a non-coaxial technique. Post-procedural monitoring included renal ultrasonography performed immediately after the biopsy and on the following day, both of which demonstrated no evidence of hematoma formation. Furthermore, laboratory evaluation on the day after the procedure confirmed no significant decline in hemoglobin levels. Six days after the biopsy, the patient experienced sudden, severe right lower quadrant abdominal pain, rapid hypotension, and prominent abdominal distension. An urgent contrast-enhanced CT identified active retroperitoneal bleeding originating from the right lumbar artery, leading to a rise in bladder pressure to 35 mmHg (normal range: 5-8 mmHg) and rapid progression to ACS (Figure 2).

**Figure 3:**
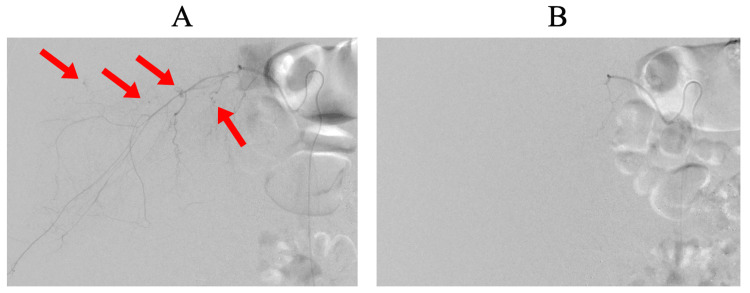
IVR embolization for right lumbar artery bleeding (A) The right lumbar artery before IVR showing contrast extravasation (red arrows). (B) The right lumbar artery after IVR with extravasation successfully resolved. IVR, interventional radiology.

Due to ongoing hemodynamic instability, immediate IVR embolization of the right lumbar artery was undertaken using 25% n-butyl-2-cyanoacrylate as the embolic agent (Figure 3).

**Figure 4:**
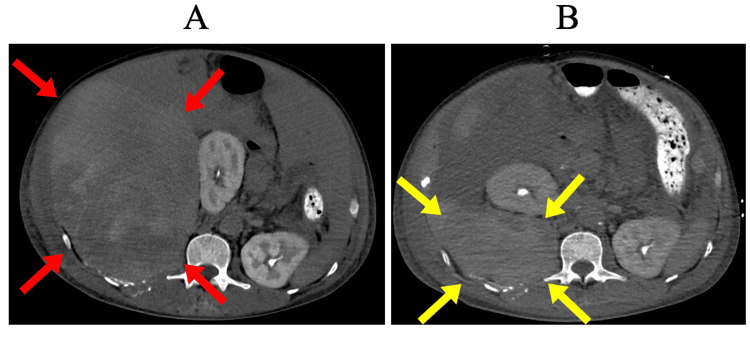
Surgical removal of retroperitoneal hematoma (A) Preoperative retroperitoneal hematoma (red arrows). (B) Postoperative reduction of the retroperitoneal hematoma (yellow arrows).

Despite initial control, bleeding recurred twice from different branches of the lumbar artery, necessitating two additional IVR procedures over the subsequent 48 hours. Notably, extravasation was observed from newly affected branches, which could not all be attributed to direct injury from the renal biopsy. We hypothesize that progressive enlargement of the retroperitoneal hematoma may have led to secondary bleeding from previously uninjured branches of the lumbar artery. After achieving stable hemostasis, an urgent surgical evacuation of the retroperitoneal hematoma was conducted, significantly reducing intra-abdominal pressure (Figure 4).

**Figure 5:**
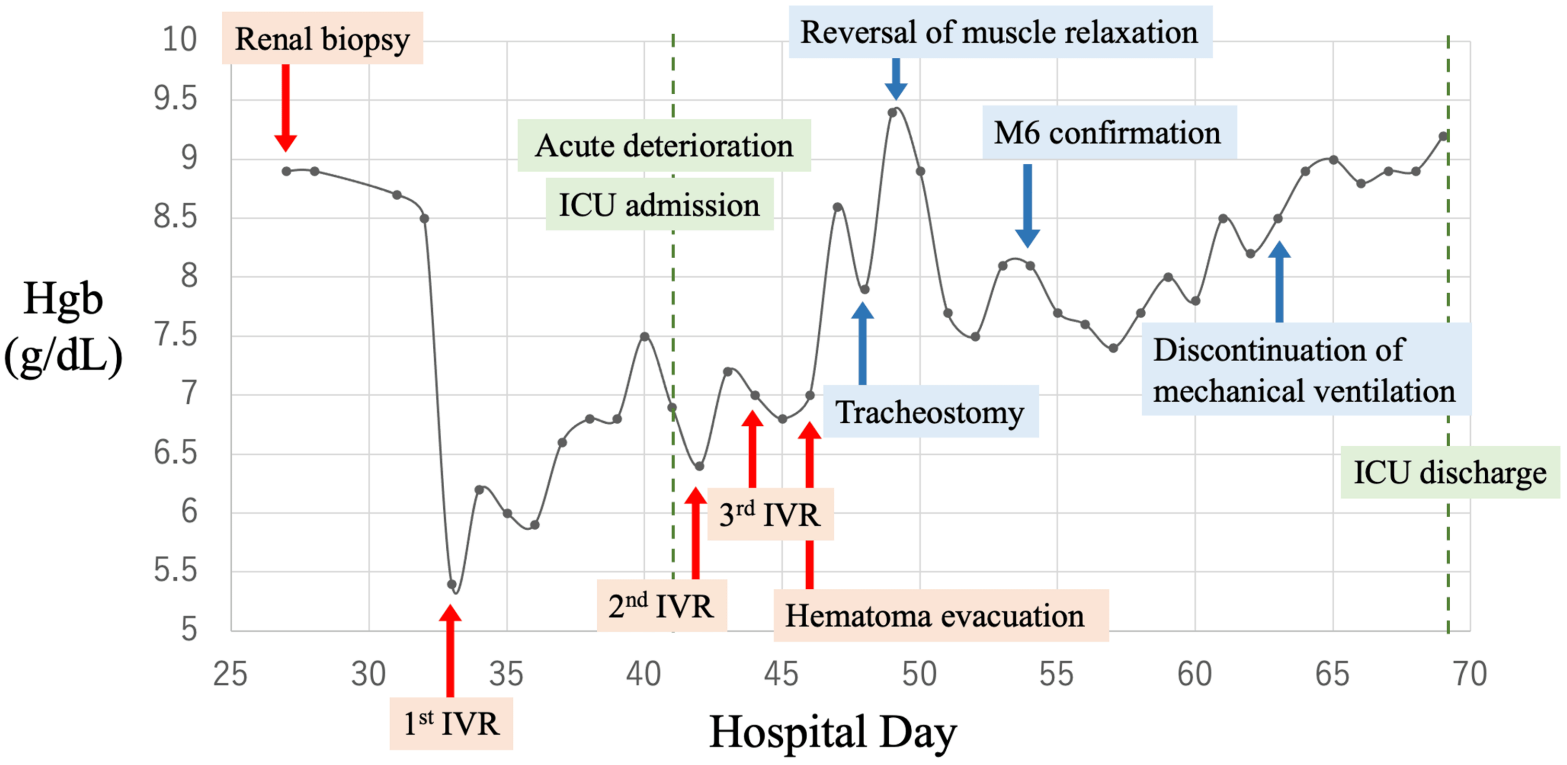
The clinical course of this case A renal biopsy was performed on day 27. On day 33, a delayed retroperitoneal hemorrhage was detected, and the first IVR was performed. The patient experienced sudden deterioration on day 41 and was admitted to the ICU. The second IVR was performed on day 42, followed by the third IVR on day 44. On day 46, a hematoma evacuation surgery was performed. No further rebleeding occurred, and the patient was discharged from the ICU on day 69. IVR, interventional radiology.

Postoperatively, the patient’s condition stabilized rapidly, with intra-abdominal pressure normalizing to 7 mmHg. He was successfully weaned from mechanical ventilation, with no further bleeding or complications noted during hospitalization. As his condition stabilized, he was transferred to a general ward on day 68 (Figure 5).

## Discussion

This case highlights the critical importance of early diagnosis and timely intervention in managing ACS secondary to retroperitoneal hemorrhage following renal biopsy, particularly in patients with underlying coagulopathy. Given the potential for rapid clinical deterioration, prompt recognition and proactive therapeutic action are essential to minimize the risk of multiorgan dysfunction and irreversible organ damage [6].

One key insight from this case is that the timing of surgical decompression following IVR embolization plays a crucial role in patient outcomes. Early surgical intervention after successful embolization enabled rapid reduction of intra-abdominal pressure, which was likely instrumental in preventing further organ failure. This suggests that prompt surgical evacuation of hematomas, when performed after securing hemostasis through IVR, may be beneficial in improving patient prognosis.

However, the optimal timing for hemostatic interventions, including both IVR embolization and subsequent surgical management, remains an area of ongoing debate. There is still no clear consensus on the best strategy for managing post-biopsy hemorrhage, and individualized treatment approaches are required based on each patient’s condition [7].

Although hematoma evacuation is not routinely indicated in most cases, its implementation after successful IVR embolization may offer additional clinical advantages [8]. In particular, early reduction of intra-abdominal pressure can facilitate faster weaning from mechanical ventilation and minimize the duration of deep sedation, which is especially beneficial in critically ill patients. While such an approach is not yet included in current consensus statements, it may be a valuable option to consider in selected high-risk patients.

Future clinical research is needed to establish evidence-based protocols for personalized management strategies, particularly for high-risk patients with coagulopathies or other comorbidities. The integration of multidisciplinary expertise, including surgeons, interventional radiologists, intensivists, and nephrologists, is essential to optimize patient care and improve outcomes in such complex and rare cases. As demonstrated in this report, close coordination among multiple specialties is indispensable in successfully managing ACS secondary to biopsy-related hemorrhage.

## Conclusions

This case emphasizes critical aspects of successful management, including early diagnosis, multidisciplinary collaboration, and individualized therapeutic strategies, in managing ACS resulting from delayed retroperitoneal hemorrhage post-renal biopsy. Prompt IVR embolization combined with timely surgical hematoma evacuation appeared beneficial for favorable outcomes, reinforcing the significance of proactive clinical decision-making, especially in patients with heightened bleeding risks due to coagulopathies or liver dysfunction. Further research is required to establish standardized management protocols for ACS in patients with biopsy-related hemorrhagic complications.
